# Biomass Chitosan-Based Tubular/Sheet Superhydrophobic Aerogels Enable Efficient Oil/Water Separation

**DOI:** 10.3390/gels9040346

**Published:** 2023-04-18

**Authors:** Wenhui Wang, Jia-Horng Lin, Jiali Guo, Rui Sun, Guangting Han, Fudi Peng, Shan Chi, Ting Dong

**Affiliations:** 1 College of Textile and Clothing, Qingdao University, 308, Ningxia Road, Qingdao 266071, China; 2 Advanced Medical Care and Protection Technology Research Center, Qingdao University, 308 Ningxia Road, Qingdao 266071, China; 3 Advanced Medical Care and Protection Technology Research Center, Department of Fiber and Composite Materials, Feng Chia University, Taichung City 407102, Taiwan; 4 School of Chinese Medicine, China Medical University, Taichung City 404333, Taiwan; 5 Key Laboratory of Bio-Fibers and Eco-Textiles, Qingdao University, 308 Ningxia Road, Qingdao 266071, China; 6 Fujian Aton Advanced Materials Science and Technology Co., Ltd., Fujian 350304, China; 7 Bestee Material Co., Ltd., Qingdao 266001, China

**Keywords:** chitosan, poplar catkin fiber, superhydrophobic aerogels, layered tubular/sheet structures, oil/water separation

## Abstract

Water pollution, which is caused by leakage of oily substances, has been recognized as one of the most serious global environmental pollutions endangering the ecosystem. High-quality porous materials with superwettability, which are typically constructed in the form of aerogels, hold huge potential in the field of adsorption and removal of oily substances form water. Herein, we developed a facile strategy to fabricate a novel biomass absorbent with a layered tubular/sheet structure for efficient oil/water separation. The aerogels were fabricated by assembling hollow poplar catkin fiber into chitosan sheets using a directional freeze-drying method. The obtained aerogels were further wrapped with -CH_3_-ended siloxane structures using CH_3_SiCl_3_. This superhydrophobic aerogel (CA ≈ 154 ± 0.4°) could rapidly trap and remove oils from water with a large sorption range of 33.06–73.22 g/g. The aerogel facilitated stable oil recovery (90.07–92.34%) by squeezing after 10 sorption-desorption cycles because of its mechanical robustness (91.76% strain remaining after 50 compress-release cycles). The novel design, low cost, and sustainability of the aerogel provide an efficient and environmentally friendly solution for handling oil spills.

## 1. Introduction

Gasoline is increasingly in demand because of recent industrial developments, yet leakage of oily substances during the processes of exploitation and transportation becomes a dire consequence [1,2]. For example, the outbreak of the Gulf War in 1991 resulted in a leak of oil of about 1.5 million tons, forming an oil band that was 16 km long and 3 km wide near Saudi Arabia at a spreading rate of 24 km/day. Unfortunately, any minor leakage of oil negatively affects the marine ecological system in various ways [3]. For example, the frequency of marine red tide is in direct proportion to the content and frequency of oil leakage [4,5]. Oil leakage over oceans may also adversely affect the health of infants and toddlers, potentially inflicting them with asthma and a higher death toll, of which the results may take shape over numerous years. As a result, the removal of leaked oils over the sea becomes a worldwide concern, and traditional management involves physical, chemical, and biological methods [6,7]. The physical methods, such as mechanical skimmers, have the disadvantages of costly oil/water separation machines and tremendous consumption of both manpower and material resources. Chemical methods include chemical dispersants and in situ burning, of which the former involves spraying a dispersion agent containing a toxic substance, while the latter generates tremendous toxic gases, causing secondary pollution and energy waste [8]. Biological methods rely on microorganisms to decompose leaked oils. However, these methods are only suitable for small-scale oil leakage. By contrast, the sorption-based method has been regarded as an energy-saving approach for oil contaminant disposal because of low production cost and low demand for manpower.

The oil/water separation efficiency via absorption is dependent on the hydrophilic/hydrophobic attributes and micro-pore structure of the absorbent. In particular, high-quality porous materials with superwettability, which are typically constructed in the forms of aerogels, hold huge potential in the field of adsorption and removal of oily substances form water because of their abundant and tunable porous structure, lightweight feature, and programmable surface groups. To date, a large number of aerogels have been developed, including the magnetic superhydrophobic melamine sponge (oil absorption ratio: 39.8–78.7 g/g) [9], polyurethane foam coated with polysiloxane-modified clay nanotubes (oil absorption ratio: 20–105 g/g) [10], graphene-coated carbon nanofiber (G-CNF) foam (oil absorption ratio: 86–153 g/g) [11], carbon nanotube sponges (oil absorption ratio: 80–180 g/g) [12], graphene/PDMS sponge (oil absorption ratio: 4.2–13.7 g/g) [13], and graphene/nanofiber aerogels (oil absorption ratio: 230–734 g/g) [14]. At present, the majority of aerogels are composed of synthetic materials that are favorable for mass production, and their waste either cannot be decomposed or has a very small decomposition rate, leading to severe secondary pollution. Carbon-based aerogels have the advantage of excellent oil absorption capacity, yet their fabrication demands tremendous energy and resources, which makes their mass production impossible. For example, carbon nanofiber (CNF) aerogels need to extract nano-cellulose from plants. However, the original plant fibers contain drastic hydrogen bonding between the fibrous groups, which means the extraction of nano-cellulose requires enzyme catalysis or chemical pre-treatment followed by mechanical decomposition. Compared to synthetic materials and carbon-based materials, such as natural fiber (kapok fiber [15,16], cotton fiber [17], populus seed fibers [18], kenaf core fiber, milkweed floss [19], etc.), they feature abundant natural resources, a low production cost, and biodegradation. Biomass aerogels are usually produced via freeze-drying in pace with superhydrophobic modification via the principle of constructing low surface energy and hierarchically rough surface [20]. At present, the majority of biomass-based materials lack sufficient mechanical endurance, and thus the oil absorption performance is compromised after multiple squeeze cycles. Moreover, due to the intrinsic fragility and internal chaotic porous structure, biomass materials are prone to have a collapse of internal structure and a swelling-then-dissolution feature when used in a water environment, which in turn severely restricts the biomass aerogel from the oil-water separation application. In nature, some natural materials like seaweed, lotus stems, and wood show an orderly, organized interior and a regular structure, hence demonstrating extraordinary mechanical properties [21,22]. Derived from conventional freeze-drying techniques, directional freeze-drying technology generates a temperature gradient in a single direction, which promotes the directional growth of ice crystals in the precursor to form an oriented porous structure along the temperature gradient after freeze-drying [23,24,25]. This method become an efficient method of improving the mechanical compression performance of aerogels, such as a high-strength (CNF)/polyvinyl alcohol (PVA)/graphene oxide (GO) aerogel showing an anisotropic porous structure [26] and a wood-inspired elastic biomass aerogel with special spring-like morphology [27].

On the other hand, environmental concerns regarding discarded solid debris containing oil residues due to the indiscriminate use of various sorption materials are often overlooked. These discarded oil-containing materials, which take up lots of land, have led to global environmental concerns regarding the release of microplastics due to the long-term environmental weathering [28,29]. For example, synthetic polymer absorbents, such as PU sponges, PS sponges, PP nonwovens, and PET nonwovens, which are nondegradable, have been proved to be the main contributor of detected microplastics in the terrestrial soil. The occurrence of microplastics in the terrestrial soil will cause hetero- or homo-aggregation with various microorganisms and macromolecules and disturb the vital aspects of soil like soil colloids and soil micro flora and fauna [30]. Biomass materials are biodegradable and environmentally friendly, but many biomass-based aerogels, which are generally fabricated by freeze-drying in pace with chemical cross-linkage to obtain the necessary strength, are less biodegradable [18]. Therefore, it is of global significance to develop sustainable alternatives combining high oil sorption performance, excellent biodegradation, and good reusability as oil absorbents.

Poplar is a common wood that grows worldwide. Its fruits grow ripe in the spring, and the cracked fruits produce numerous catkin fibers (PC) flying around in the air, which generally causes environmental pollution, possible fires, and allergic reactions in people. PC fibers show a unique hollow structure, and their composition enables good liquid adsorption capacity. Chitosan (CS), which is a linear polymer of β-(1 → 4)-linked 2-acetylamino-2-deoxy-β-D-glucopylanopylanoid and 2-amino-2-2 deoxy-β-d-glucopylanoid, is mainly extracted from crab/shrimp shells and obtained through the deacetylation of chitin. The degree of deacetylation of commercial CS is generally above 60%. As low-cost, biodegradable, non-toxic, and biocompatible, CS has been reported to be widely used in food additives, drug release, oil adsorption, heavy metal adsorption, and tissue engineering scaffolds [31,32,33]. Herein, we developed a facile strategy to fabricate a novel biomass absorbent with a layered tubular/sheet structure by a directional freeze-drying method, through which hollow PC fiber was assembled into chitosan sheets. The obtained aerogels were further wrapped with -CH_3_-ended siloxane structures through a facile chemical vapor deposition (CVD) process using CH_3_SiCl_3_. This aerogel was used as an oil sorbent to efficiently trap and remove oils from water. The aerogels also showed mechanical robustness, which facilitated stable oil recovery for repeated oil/water separation by squeezing. The novel design, low cost, and sustainability of the sorbent reported here provides an efficient and environmentally friendly solution for the handling of oil spills.

## 2. Results and Discussion

**Figure 1:**
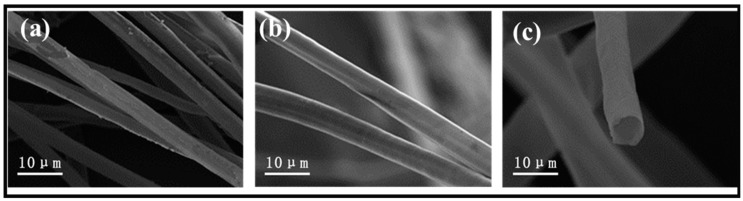
(**a**) SEM image of non-treated PC and (**b**,**c**) SEM images of treated PC.

**Figure 2:**
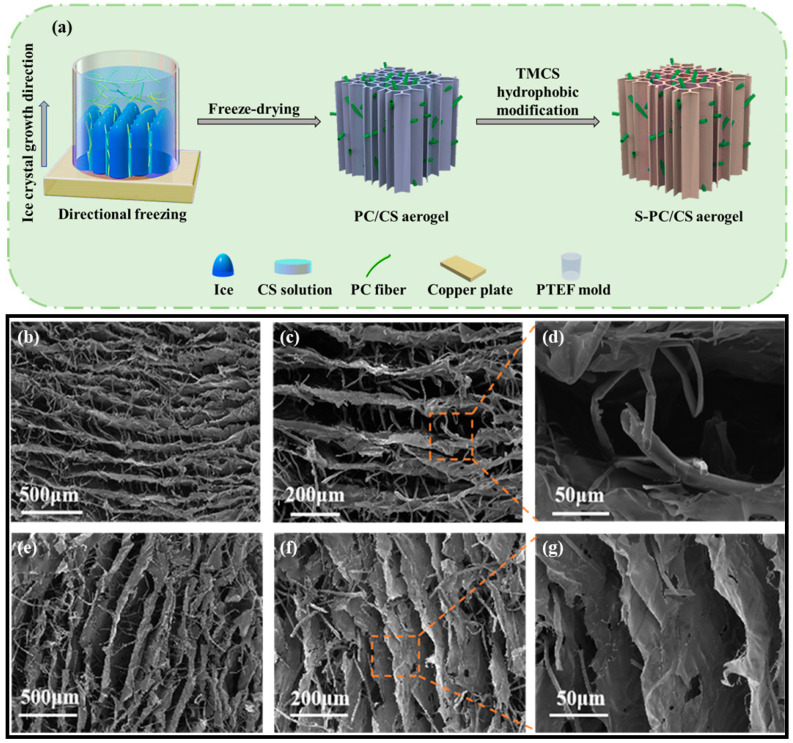
(**a**) Manufacturing process of S-PC/CS aerogel; (**b**–**d**) horizontally cutting section and (**e**–**g**) vertically cutting section of S-PC/CS aerogel.

The PC fibers were collected from the Qingdao University of Shandong province in China and were highly hollow, with a fiber wall thickness of 330 nm and an inner diameter of 6.63 μm, meaning the hollow part took up 90.7% of the total volume (Figure S1). On top of the hollow structure, the wax layer also provided PC fibers with hydrophobic features. In this study, to scatter the PC fibers in the water, the wax over the surface was first removed. Figure 1a–c compares the stereomicroscopic and SEM images of the PC fibers, and the treated PC fibers have a sleeker surface while retaining their hollow structure. The results suggest that pre-treatment does not change the intrinsic structure or features of PC fibers. The treated PC fibers and the CS (as a thickening agent) were mixed to form a stabilized suspension. The suspension was poured into a PTEF mold that was connected to a copper plate and placed in a freezer. As a result, ice crystals grew along a specified direction, and eventually the longer PC fibers became curly and entangled. Meanwhile, the CS became the connective points among the fibers, and after the crystals were removed via the freeze-drying process, there was an initiating configuration of PC/CS aerogel (Figure 2a). In the aerogel, PC fibers that had a hollow structure were assembled into chitosan sheets showing a layered tubular/sheet structure (Figure 1a–c). PC fibers that can be used as a second-pore capillary have a positive influence over the oil transport of aerogel, while the CS serves as bonding points that make the aerogel mechanically robust. The resulting tubular/sheet structure has a sheet structure (from freeze-drying the CS) as the first gradient of oil absorption as well as a concurrent hollow structure (from the PC fibers) as the second gradient of oil transport. As a result, the aerogel demonstrates a highly strengthened oil sorption capacity that is guaranteed by its super high porosity and lower volume density (0.011 g/cm^3^).

**Figure 3:**
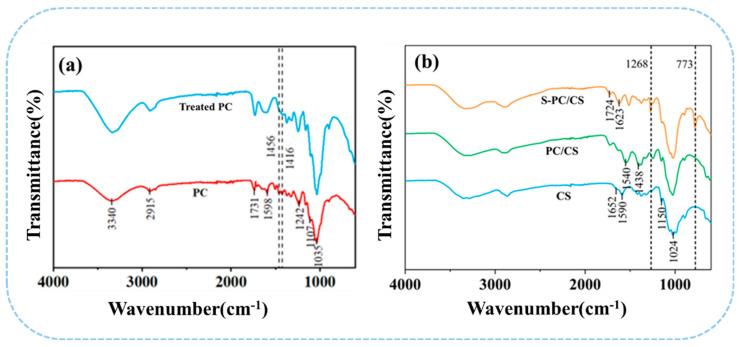
FTIR spectra of (**a**) treated/non-treated PC fibers as well as (**b**) CS, PC/CS, and S−PC/CS aerogel.

As seen in Figure 3a,b, FTIR spectra is mainly used to study the differences in the surface functional group of non-treated PC fibers and the S-PC/CS aerogel. PC fibers, as cellulose fibers, contain many characteristic function groups, such as -OH (3340 cm^−1^), C-H (2921 cm^−1^), C-O (1737 cm^−1^ and 1237 cm^−1^), C=C (1590 cm^−1^), C-O-C (1106 cm^−1^), and C-O (1037 cm^−1^) [18]. By contrast, the treated PC fibers demonstrate significantly attenuated bands at 1598 cm^−1^, 1242 cm^−1^, and 1456 cm^−1^ that corresponded to the tensile vibration of the aromatic C skeleton, which confirms that the wax is removed from the PC fibers. Moreover, after the superhydrophobic treatment, S-PC/CS aerogel exhibits characteristic peaks at 773 cm^−1^ corresponding to the Si-O-Si bond and an asymmetric stretching band at 1271 cm^−1^ corresponding to the C-Si-O group. The Si-O bond indicates that the derived -OH from PC/CS has a drastic interaction with organosilane, which in turn forms a silicon-oxygen bond over the surface of the PC/CS aerogel, suggesting a chemical reaction between TMCS and the PC/CS aerogel. The peaks of the hydroxyl group in the S-PC/CS are still present. This is because the connection between MTCS and material is relatively complicated. During the process, MTCS undergoes self-polymerization with H_2_O vapor to produce 3D methylsiloxanes with reactive trifunctional silanes that can bond with hydroxyl groups on the fiber surface via -Si(OH)_3_ radicals. However, due to steric hindrance, these surfaces should contain “holes” between randomly attached disiloxane groups that are smaller than the disiloxane, contain surface hydroxyl groups, and cannot be filled by further reaction (Figure S2) [34].

**Figure 4:**
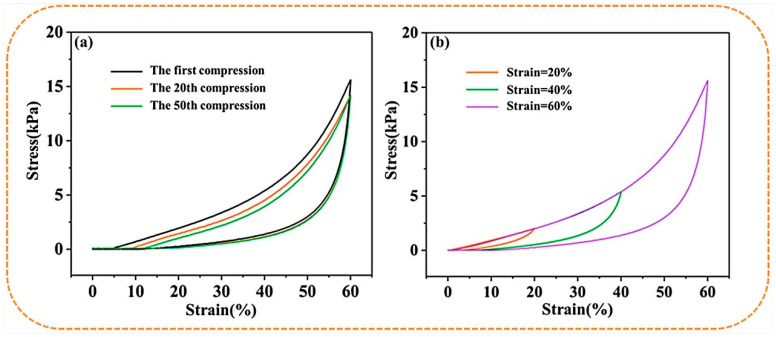
The stress-strain curves of S−PC/CS aerogel as related to (**a**) constant 60% strain and (**b**) various strains.

Figure 4a,b shows the cyclic compression performance of the S-PC/CS aerogel tested with a constant 20–60% strain. The deformation behavior of the S-PC/CS aerogel includes the linear elastic area when ε < 20%, the subsequent plateau stage when 20% < ε < 40%, and the densification stage when the stress accelerates. With a compression strain (ε = 40%), the S-PC/CS aerogel demonstrates marginal plastic deformation (6.81%) and a height recovery rate of 93.19%. Furthermore, when a greater compression stress (ε = 60%) is exerted, the S-PC/CS aerogel presents a plastic deformation of 13.23%. The test results also indicate that the aerogel exhibits excellent fatigue resistance. The produced plastic deformations are 4.83% after the first cycle and 8.57% after 20 cycles. Nonetheless, the effects of multiple plastic deformations are accumulated, and the aerogel at the 50th cycle of loading-unloading is inflicted with irreversible damage of 11.29%. To data, a majority of biomass aerogels still present crucial limitations on structural and mechanical stability. As illustrated in Table 1, the existing biomass aerogels have a maximum compressive stress of 3.5–55 kPa, and many of them have the problem of structural instability and plastic deformation exceeding 15%, such as dialdehyde carboxymethyl cellulose aerogels (<10 kPa, 15–20% plastic deformation after 50 cycles) [35], cellulose nanocrystals/PVA aerogels (<35 kPa, >15% plastic deformation after 50 cycles) [36], cellulose nanofibrils/N-alkylated chitosan/poly(vinyl alcohol) aerogels (<55 kPa, but the maximum stress will reduce to 17 kPa after 50 cycles, 18–20% plastic deformation) [37], seed hairs of typha orientalis aerogels (<25 kPa, 14.8% plastic deformation after 10 cycles) [38], and so on. The excellent rebound property of the S-PC/CS is primarily attributed to the unique sheet structure. When the aerogel is compressed, the sheet structure provides enough space for elastic deformation while saving energy. When the external force is withdrawn, the energy in need is released, which allows the aerogel to recover its original state. Meanwhile, the PC fibers among the sheets of aerogel also provide a proportion of support, which benefits the compression strength of the aerogel. The excellent mechanical properties and compression recovery prove that the materials can be repetitively used.

**Figure 5:**
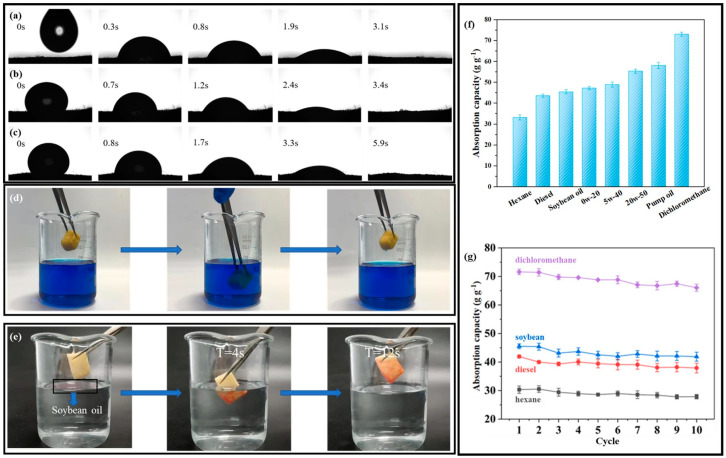
The images of S−PC/CS absorbing (**a**) soybean, (**b**) motor oil 0W−20, and (**c**) motor oil 5W−40. (**d**,**e**) Hydrophobic performance test. (**f**) Sorption capacity (as related to the liquid) and (**g**) Sorption capacity as related to the liquid and the multiple cycles.

Figure 5a–c shows the effects of three types of oil on the S-PC/CS aerogel, and the difference in the speed of infiltrating the aerogel among the different oils is ascribed to the viscosity and mobility. It takes the aerogel only 3.1 s to absorb vegetable oil completely, 5.9 s for viscous motor oil ^1#^ 5W−40, and 3.4 s for viscous motor oil ^2#^ 0W−20, which substantiates that the modified aerogel exhibits excellent oil sorption rate regardless of the oil type. In addition, the original PC/CS is highly hydrophilic, and the modification of CH_3_SiCl_3_ results in the replacement of the hydroxyl groups of the materials by -CH_3_-ended siloxane structures. This process effectively transfers the hydrophobic properties of the aerogels with an average WCA of about 154 ± 0.4º which is highly hydrophobic and satisfies the requirement of oil/water separation. In order to test the hydrophobicity and selective adsorption of the aerogel, the following tests were performed. As shown in Figure 5d, the aerogel was put into water stained with methylene blue for a period of time and taken out, and it was found that the aerogel was not dyed. As shown in Figure 5e, a few drops of soybean oil dyed with oil red O were dropped on the water surface, and then the aerogel was immersed in water. After a period of time, the soybean oil on the water surface was completely adsorbed by the aerogel, which proves that the aerogel has good hydrophobicity and selective adsorption of oil. Different liquids are employed to examine the oil sorption capacity of the aerogel. Figure 5f shows that the aerogel demonstrates the maximal and minimal sorption capacity for dichloromethane and hexane, respectively. Furthermore, the sorption capacity is ascending for diesel, soybean oil, motor oil ^1#^, motor oil ^2#^, motor oil^3#^ 20W-50, and pump oil in a range of 33.06–73.22 g/g^−1^, and the corresponding sorption capacity is dependent on the density of the liquids. In addition, the repetitive use of aerogel was tested, as seen in Figure 5g. An aerogel was immersed in the test oil for 10 minutes and then placed over a filter for another 1 min to remove redundant oil. After 10 cycles of sorption-desorption, the sorption capacity of the aerogel was decreased by 5.48 g/g for dichloromethane, 4.51 g/g for soybean, 4.10 g/g for diesel, and 2.69 g/g for hexane. During the 10 cycles of sorption-desorption, the aerogels retained 90.07–92.34% of their initial sorption capacity. The oil sorption tests of the aerogels under 50 sorption-desorption cycles were also carried out using soybean oil. As shown in Figure S3a, the sorption capacity of aerogel was decreased by 0.985 g/g after 20 cycles, 1.378 g/g after 30 cycles, 1.15 g/g after 40 cycles, and 0.81 g/g after 50 cycles. During the 50 cycles of sorption-desorption, the aerogels retained over 85.6% of their initial sorption capacity. In other words, the oil sorption capacity of the aerogel was not significantly compromised by the test, which suggests that the aerogel can be repetitively used and is an ideal oil/water separation material. In addition, the oil/water selectivity performance in Figure S3b shows that the aerogel retained a high oil sorption capacity of 47.48–44.49 g/g in 10 testing cycles, while the water sorption capacity was very low and in the range of 0.002–0.020 g/g, indicating a high oil/water selectivity of 222.45–19,869.46. The above characteristics feature S-PC/CS aerogel as a great application prospect in controlling oil spills. Table 1 compares the S-PC/CS with a wide range of current state-of-the-art biomass-based oil absorbents. Compared with biomass aerogels, which are generally derived from cellulose, chitosan, sodium alginate, lignin, etc., the S-PC/CS outperforms the majority of aerogels in terms of oil sorption capacity and hydrophobicity, such as dialdehyde carboxymethyl cellulose aerogels (sorption capacity: 20–30 g/g, WCA = 144.5º) [35], graphene ox-ide/halloysite nanotubes (RGO/HNTs) membrane (WCA = 82.43º) [39], chitin/halloysite nanotubes sponges (sorption capacity: 11.23 g/g, WCA = 88–98º) [40], HNTC-FG-PU sponges (sorption capacity: 50.8 g/g, WCA = 145 ± 2º) [41], and alginate/oil gelator aerogels (sorption capacity: 32 g/g, WCA = 155 ± 5º) [42]. The results indicate that the tubular/sheet structure facilitates fast oil transport. The aerogel contains chitosan that forms narrow channels in tidy alignment, which provides sufficient space for oil transmission. Moreover, the tubular structure of PC fibers serves as a second channel that expedites the infusion of oil, and thus the aerogel demonstrates excellent oil sorption performance. At the same time, the WCA of the aerogel modified by super hydrophobicity is better than most aerogels. The excellent hydrophobicity endows the aerogel with the characteristic of selective adsorption. In addition, the raw material of aerogel comes from pure biomass material, which is low cost and easy to obtain, biodegradable, and environmentally friendly. These attractive advantages of the aerogel give it a broad application prospect, and it is expected to be used in industrial wastewater treatment and marine oil and water separation.

**Table 1:** Comparison of properties of different sorption materials.

Compressive Properties							
Sorbent Material	Maximum Compression Stress (kPa)	Number of Compression Cycles	Plastic Deformation	WCA	Sorption Capacity (g/g^−1^)	Preparation Method	Reference
Lignin/Agarose/PVA aerogels	<16	10	20%	150º	18	Indirectional freeze-drying	Jiang, J., et al., 2017 [43]
Dialdehyde carboxymethyl cellulose aerogels	<10	50	15–20%	144.5º	20–30	Indirectional freeze-drying	Zhang, F., et al., 2022 [35]
Cellulose nanocrystals/PVA aerogels	<35	50	>15%	136º	<35	Indirectional freeze-drying	Gong, X., et al., 2019 [36]
Carboxylated cellulose nanofibers/PEI aerogels	<9	1	20%	-	20–60	Indirectional freeze-drying	Tang, R., et al., 2023 [37]
Cellulose nanofibrils/N-alkylated chitosan/poly(vinyl alcohol) aerogels	<55 kPa	50	18–20%	147º	19–51	Indirectional freeze-drying	Li, M., et al., 2021 [44]
Seed hairs of typha orientalis aerogels	<25	10	14.8%	153º	42–160	Carbonized	Yang, J., et al., 2018 [38]
Bacterial cellulose aerogels	<3.5	100	5%	131 ± 3.5º	37–89	Pyrolysis	Ieamviteevanich, P., et al., 2020 [45]
Alginate/oil gelator aerogels	<9 kPa	-	-	155 ± 5º	32	Indirectional freeze-drying	Wang, Y., et al., 2022 [42]
graphene oxide/halloysite nanotubes (RGO/HNTs) membrane	-	-		82.43º	-	Hummers method	Liu Y., et al., 2018 [39]
chitin/halloysite nanotubes sponge	-	-	-	88–98º	11.23	Freeze-drying	Zhao X, et al., 2019 [40]
HNTC-FG-PU sponges	-	-	-	145 ± 2º	50.8	Dip-coating	Prasanthi, I., et al., 2022 [41]
S-PC/CS aerogels	16.5 (ε = 60%)	50	8.25%	154 ± 0.4º	33.06–73.22	YES	This work

## 3. Conclusions

In this study, chitosan is used as the basic material and is combined with PC fibers to form a CS-based aerogel with a unique tubular/sheet structure that mechanically improves the biomass aerogel. After 50 cycles of a compression resistance test, the aerogel only exhibits marginal irreversible deformation (8.24%). The aerogel exhibits an oil sorption range of 33.06–73.22 g g^−1^, which outperforms the majority of recently reported sponges. The multiple sorption-desorption results indicate that the aerogel retains a stable oil sorption capacity that is over 90%, suggesting that the aerogel can be repetitively used. As the proposed aerogel is pure biomass material, it can be decomposed in nature and is eco-friendly. Also, the needed raw material for the chitosan-based pure biomass aerogel is easily accessible, and the aerogel can be expected to be used in oil/water separation on a large scale compared to the conventional aerogel.

## 4. Material and Methods

### 4.1. Materials

The PC fibers (hereafter referred to as PC fibers) were collected from poplar trees at Qingdao University, Shandong. Chitosan (CS) (95% degree of deacetylation, 100–200 mPa·s) was purchased from Aladdin, Industrial Co., Ltd., Shanghai, China. Hexane, absolute alcohol, acetic acid, dichloromethane, chloroform, and ethyl acetate were purchased commercially without further purification. Methyltrichlorosilane (MTCS, 98%) was obtained from Sigma, America. Methylene blue and oil red O were both from Hefei Sifu Biotechnology Co., Ltd., Hefei, China. Several oils were purchased commercially (see Table S1).

### 4.2. Pre-Treatment of PC Fibers

The PC fibers were trimmed to a length of 5–10 mm and rinsed with deionized water and ethanol several times in order to remove any impurities from the surface. Next, NaClO_2_ (3 g) was dissolved in 300 mL of deionized water, after which 0.9 mL of acetic acid and 3.0 g of PC fibers were added to be heated in a water bath at 75 °C for 2.5 h. Afterwards, PC fibers were filtrated and once again rinsed with deionized water until the residual liquid becomes neutral. The fibers were removed and dried in an oven at 70 °C for 12 h.

### 4.3. Preparation of Aerogels

PC fibers (0.8 g) and CS (1.4 g) were added to deionized water (200 mL, 60 °C), after which 2 mL of acetic acid (1%, *v*/*v*) was added. The blends were mixed using a household blender to form suspensions with uniform quality. Next, the suspension was infused into a PTFE mold, with the bottom of the mold in contact with a copper plate, and was then frozen in liquid nitrogen. After being totally frozen, the suspension underwent the freeze-drying process for 48 h, resulting in the aerogel (hereafter referred to as PC/CS).

### 4.4. Modification of Aerogels

The PC/CS aerogel was placed in an environment at a humidity of 60–70% for 24 h. Next, the aerogel was placed in sealed glass, where 0.2 mL of TMCS was added and left for a 12-h reaction. The sample was removed and heated to 60 °C in an oven to remove the redundant TMCS, thereby obtaining a superhydrophobic aerogel (hereafter referred to as S-PC/CS).

### 4.5. Characterizations

A freezer dryer used (LGJ-18, Beijing Songyuan Huaxing Technology Development Co., LTD., Beijing, China) in the freeze drying process. A field emission scanning electron microscope (SEM, Zeiss Sigma500, Oberkochen, Germany) was used to observe the structure of the aerogel. The working distance and energy beam for the SEM were 3 and 5 mm, respectively, with voltage 10 keV and current 10 μA. The samples were metallized before analysis. Fourier-transform infrared spectroscopy (FTIR, Thermo Fisher, Waltham, MA, USA) was used to analyze the function groups of treated PC fibers, CS, PC/CS, and S-PC/CS in the range of 500–4000 cm^−1^. A universal material testing machine (Instron-3300, Norwood, MA, USA) was used for the compression stress-strain test with a strain rate of 20 mm/min. A drop-shaped analyzer (Theta, Biolin Corporation, Goteborg, Switzerland) was used to measure the water contact angle (WCA) of the aerogel with the specified volume of droplets being 5.0 μL. The porosity was measured by an automatic mercury intrusion porosimetry instrument (PoreMaster-33, Quantachrome, FL, USA). The volume density was calculated by the following formula:

(1) $$p=\frac{v}{m}$$

where p is the volume density, v is the aerogel volume, and m is the aerogel mass.

### 4.6. Oil Sorption Capacity

Different types of oil were dripped over the aerogel, and the time that the aerogel required to absorb the whole droplet was measured. To evaluate the oil absorption capacity, S-PC/CS aerogel was immersed in different oils and organic solvents for 10 min, and the oil-loaded aerogel was placed over a filter for one minute to remove the excess oil. After ten cycles of the absorption-squeeze test, the oil absorption capacity and reusability of samples were recorded. The oil sorption capacity was calculated according to the following formula:

(2) $$\mathrm{Oil}\;\mathrm{absorption}\;\mathrm{rate}:\;(Q)=\frac{m-m_{0}}{m_{0}}$$

where the m_0_ is for the quality of the aerogel oil absorption before, and m is for the quality of the aerogel after oil absorption. In addition, to measure the oil/water selectivity, the aerogel was completely immersed in a mixture of soybean oil/water (*v*/*v* = 1:1) for 1 h, and then the saturated aerogel was dried in the oven at 60 °C for 1 h to remove the absorbed water. The masses of absorbed water (m_w_) and oil (m_o_) were calculated using the following formulas:

m_w_ = m_1_ − m_2_. (3)

m_o_ = m_2_ − m_0_. (4)

where m_0_ is the initial mass of the gel, m_1_ is the mass of the adsorbed saturated sample, and m_2_ is the mass of the sample after removing the water. The oil/water selectivity is calculated by the ratio of oil sorption mass to the water sorption mass.

## Data Availability

Not applicable.

## Supplementary Materials

The following supporting information can be downloaded at https://www.mdpi.com/article/10.3390/gels9040346/s1: Figure S1: (a/b) Camera photos of PC trees/fibers; (c) microscopic image of PC; (d/e) microscopic image of treated PC; Figure S2: Reaction mechanism of MTCS with the material; Figure S3: (a) Adsorption capacity of soybean oil for 50 cycles, (b) Oil absorption and water absorption of aerogel in 10 cycles; Table S1: The oil used in experiments.
