# Supplementary material for: Biomass Chitosan-Based Tubular/Sheet Superhydrophobic Aerogels Enable Efficient Oil/Water Separation
Source: Gels. 2023 Apr 18;9(4):346. doi: 10.3390/gels9040346 (PMC10137560; doi:10.3390/gels9040346)
Supplement: Supplementary file 1 [file gels-09-00346-s001.zip › gels-2319615-supplementary.pdf]

Biomass Chitosan-Based Tubular/Sheet Superhydrophobic Aerogels Enable Efficient Oil/Water Separation

Wenhui Wang [1,2,†], Jia-Horng Lin [1,2,3,4,†], Jiali Guo [1,2], Rui Sun [1,2], Guangting Han [5], Fudi Peng [6], Shan Chi [7] and Ting Dong [1,2,5,*]

1 College of Textile and Clothing, Qingdao University, 308, Ningxia Road, Qingdao 266071, China
2 Advanced Medical Care and Protection Technology Research Center, Qingdao University, 308 Ningxia Road, Qingdao 266071, China
3 Advanced Medical Care and Protection Technology Research Center, Department of Fiber and Composite Materials, Feng Chia University, Taichung City 407102, Taiwan
4 School of Chinese Medicine, China Medical University, Taichung City 404333, Taiwan
5 Key Laboratory of Bio-Fibers and Eco-Textiles, Qingdao University, 308 Ningxia Road, Qingdao 266071, China
6 Fujian Aton Advanced Materials Science and Technology Co., Ltd., Fujian 350304, China
7 Bestee Material Co., Ltd., Qingdao 266001, China
* Correspondence: tingdong09@qdu.edu.cn
† These authors contributed equally to this work.

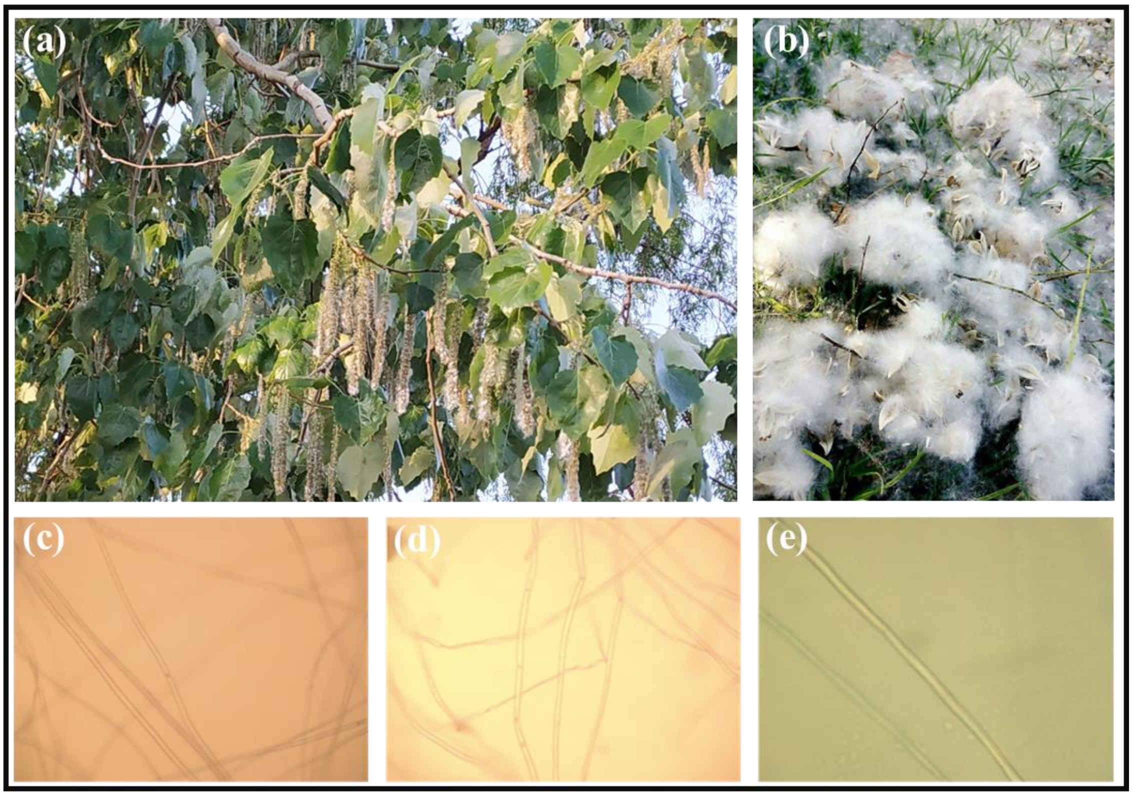

Figure S1. (a,b) Camera photos of PC trees/fibers; (c) microscopic image of PC; (d,e) microscopic image of treated PC.

Table S1. The oil used in experiments.

Oil type	Manufacturer
Diesel	China Petroleum and Chemical Corporation Limited
Soybean	Yihai Jiali Golden Arowana Grain, Oil and Food Co., LTD
Motor Oil 0W-20	Exxon Mobil Corp
Motor Oil 5W-40	Exxon Mobil Corp
Motor Oil 20W-50	Exxon Mobil Corp
Pump Oil	Leybold Optics

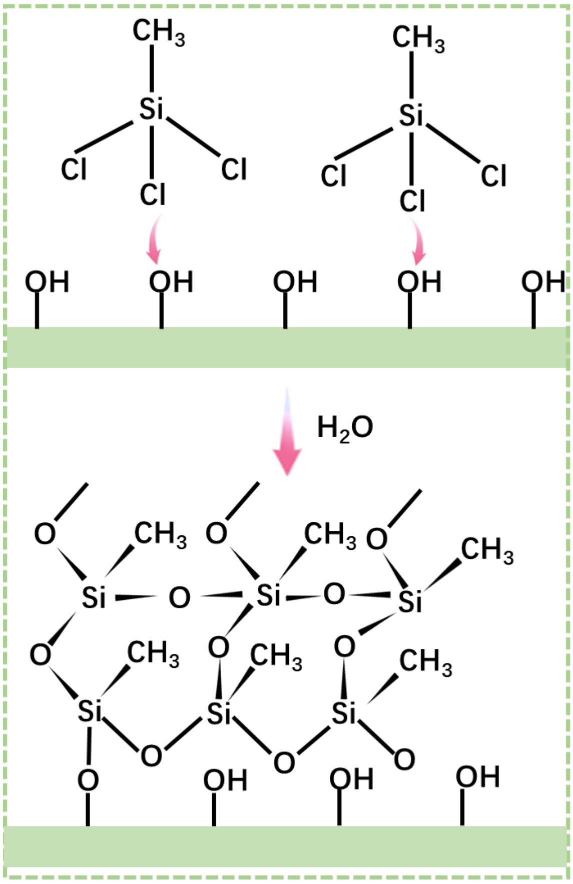

Figure S2. Reaction mechanism of MTCS with the material.

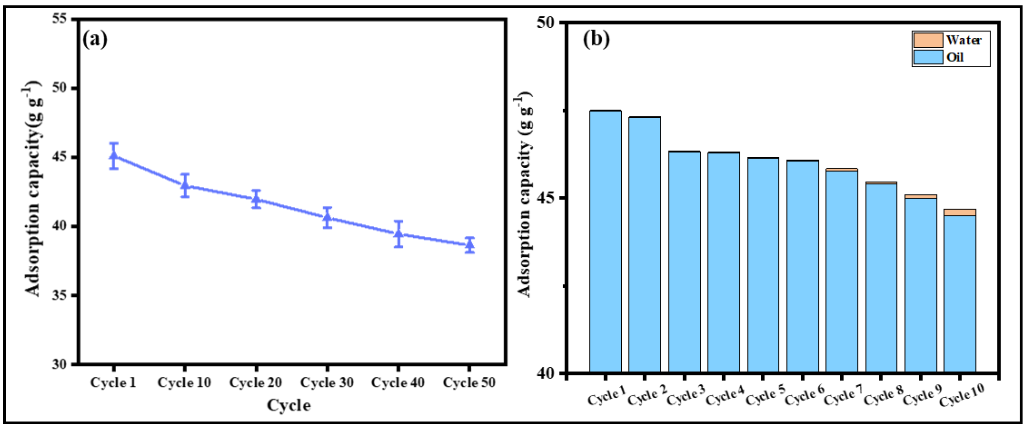

Figure S3. (a) Adsorption capacity of soybean oil for 50 cycles, (b) Oil absorption and water absorption of aerogel in 10 cycles.

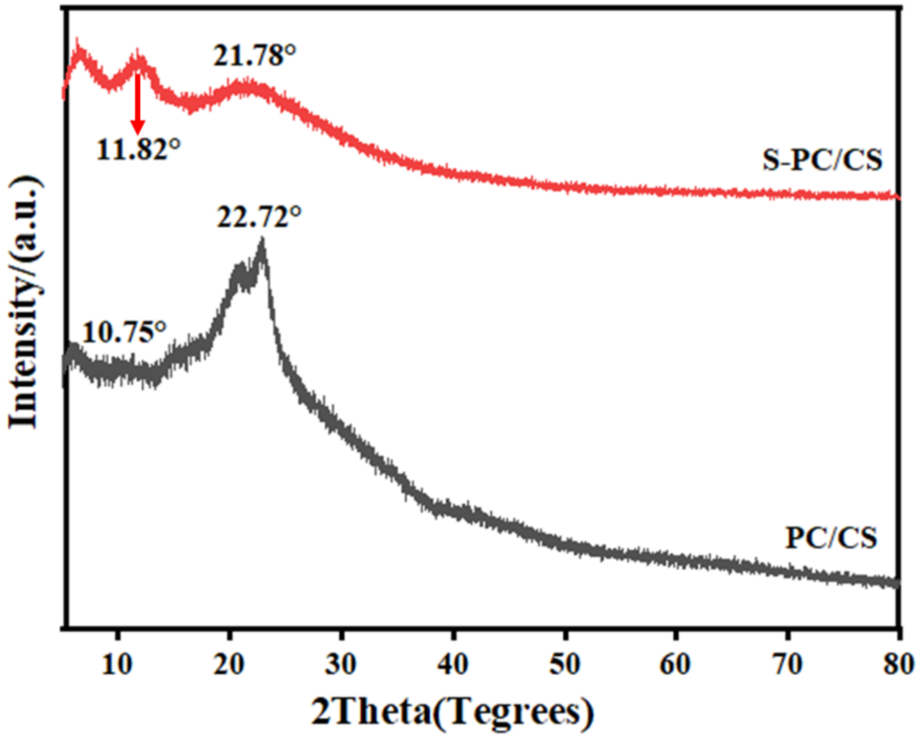

Figure S4. XRD patterns of PC/CS and S-PC/CS.
